# Surface-Engineered Li_4_Ti_5_O_12_ Nanostructures for High-Power Li-Ion Batteries

**DOI:** 10.1007/s40820-020-0366-x

**Published:** 2020-01-21

**Authors:** Binitha Gangaja, Shantikumar Nair, Dhamodaran Santhanagopalan

**Affiliations:** Centre for Nanosciences and Molecular Medicine, Amrita Vishwa Vidyapeetham, AIMS (P.O.), Kochi, 682 041 India

**Keywords:** Ultrafast charging, Li-ion battery, Lithium titanate, Off-stoichiometric synthesis, Surface chemistry

## Abstract

Materials with high-power charge–discharge capabilities are of interest to overcome the power limitations of conventional Li-ion batteries. In this study, a unique solvothermal synthesis of Li_4_Ti_5_O_12_ nanoparticles is proposed by using an off-stoichiometric precursor ratio. A Li-deficient off-stoichiometry leads to the coexistence of phase-separated crystalline nanoparticles of Li_4_Ti_5_O_12_ and TiO_2_ exhibiting reasonable high-rate performances. However, after the solvothermal process, an extended aging of the hydrolyzed solution leads to the formation of a Li_4_Ti_5_O_12_ nanoplate-like structure with a self-assembled disordered surface layer without crystalline TiO_2_. The Li_4_Ti_5_O_12_ nanoplates with the disordered surface layer deliver ultrahigh-rate performances for both charging and discharging in the range of 50–300*C* and reversible capacities of 156 and 113 mAh g^−1^ at these two rates, respectively. Furthermore, the electrode exhibits an ultrahigh-charging-rate capability up to 1200*C* (60 mAh g^−1^; discharge limited to 100*C*). Unlike previously reported high-rate half cells, we demonstrate a high-power Li-ion battery by coupling Li_4_Ti_5_O_12_ with a high-rate LiMn_2_O_4_ cathode. The full cell exhibits ultrafast charging/discharging for 140 and 12 s while retaining 97 and 66% of the anode theoretical capacity, respectively. Room- (25 °C), low- (− 10 °C), and high- (55 °C) temperature cycling data show the wide temperature operation range of the cell at a high rate of 100*C*.

**Electronic supplementary material:**

The online version of this article (10.1007/s40820-020-0366-x) contains supplementary material, which is available to authorized users.

## Highlights

- Surface-engineered Li_4_Ti_5_O_12_ nanoparticles were synthesized by an off-stoichiometric solvothermal process.
- The electrode exhibited ultrafast charge–discharge (up to 1200*C*) performances in a half-cell configuration.
- A full cell consisting of the engineered Li_4_Ti_5_O_12_ anode and LiMn_2_O_4_ cathode exhibited an ultrahigh-rate capability (up to 200*C*), long cycling life (1000 cycles), and robust performances (at − 10, 25, and 55 °C).

**Electronic supplementary material:**

The online version of this article (10.1007/s40820-020-0366-x) contains supplementary material, which is available to authorized users.

## Graphical abstract

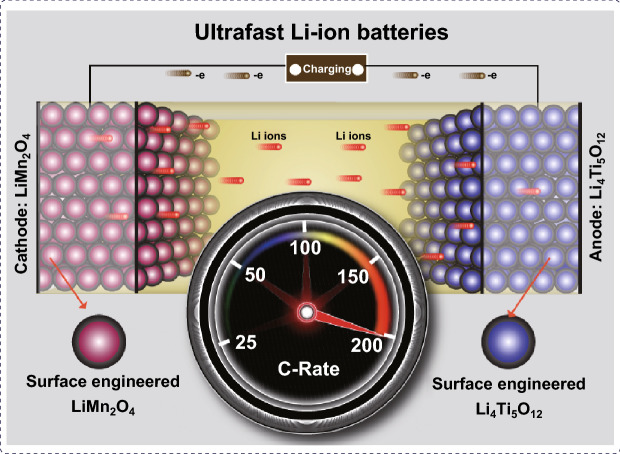

## Introduction

Fast charging is one of the important aspects of modern energy storage devices, which can enable a smooth transition from gasoline to electric vehicles without reducing the safety. Owing to its high safety and zero strain property, lithium titanate (Li_4_Ti_5_O_12_ (LTO)) has attracted significant interest as a negative-electrode material in lithium-ion batteries and capacitors [1, 2]. However, its transport properties (both ionic and electronic) are inherently limited and require significant changes for high-rate (higher than 60*C*) applications [3, 4]. The primary factors limiting the high-rate capability are the (1) lithium diffusion lengths in bulk particles, (2) poor percolated electronic conduction pathway across thick electrodes, and (3) electrode/electrolyte interface limiting the charge-transfer kinetics [5, 6]. To improve the rate capability of LTO, several strategies addressing at least one of the above three factors have been reported. For example, LTO particle size reduction to the nanoscale leads to better kinetics owing to the smaller diffusion lengths [6]. Feckl et al. [7] have reported nanoscale porous LTO thin films providing rate capabilities up to 800*C*. Nevertheless, the maximum electrode loading was only 0.14 mg cm^−2^, which limits their commercial application. Borghols et al. [8] have reported an ideal LTO particle size of approximately 30 nm to reversibly accommodate Li ions without considerable surface reconstruction. The electrical conductivity of a slurry-casted electrode can be improved by compositing large-surface-area and high-aspect-ratio carbon nanostructures as additives [9–12]. Carbon nanotubes (CNTs) as an additive can improve the rate capability of the electrode compared to the other forms of carbon [9–12]. However, it is believed that the crucial factor hindering the high-rate capabilities of electrode materials is the charge-transfer resistance at the electrode/electrolyte interface [13–17]. The electrode/electrolyte interface enabling rapid charge-transfer kinetics is regulated by the surface chemistry of the nanoparticles in the electrode. Wang et al. [14] have synthesized rutile-TiO_2_-coated LTO nanosheets providing a significant improvement in specific capacity compared to that of the uncoated counterpart. The TiO_2_-coated LTO electrode reduced the charge-transfer resistance to half of that of the uncoated sample while maintaining the good Li diffusion kinetics owing to the epitaxial growth of rutile TiO_2_ along the [001] direction. Kang et al. [17] have demonstrated the potential of surface engineering on a cathode material by synthesizing an amorphous-lithium-phosphate-coated LiFePO_4_, which provided an ultrahigh-rate capability (397*C*) with a discharge capacity of almost 60 mAh g^−1^. The rapid ionic conduction through the amorphous surface has led to such high-rate performances. Most of the studies on fast charge–discharge have demonstrated the same strategy on half-cell configurations, which limits the real-time applications. In this study, we use off-stoichiometric (Li-deficient) originators and synthesize surface-engineered lithium titanate by inhibiting the phase separation and crystallization of TiO_2_. The flawless control of the surface chemistry of LTO enables a high-power Li-ion battery with charging/discharging as fast as 12 s and 66% of the LTO’s theoretical capacity. A high-rate operation is also demonstrated in a wide temperature range of − 10 to 55 °C.

## Experimental Section

### Synthesis

For the synthesis of LTO, an off-stoichiometric (Li-deficient) proportion of lithium and titanium precursors was utilized. In a typical procedure, a molar ratio of lithium hydroxide to titanium iso-propoxide of 3.6:5 was used for the synthesis of LTO nanoparticles [18]. Initially, lithium hydroxide dissolved in distilled water was added to an ethylene glycol solvent maintained at 100 °C and was allowed to stir for 15 min. A titanium precursor was then added dropwise and allowed to stir until the formation of a clear solution. Subsequently, an ammonia solution (4.2 mL) was added. The obtained mixture was transferred to an autoclave, which was then maintained at 180 °C for 36 h in an oven. Upon completion of the 36 h solvothermal process, the obtained sample was centrifuged, washed, and annealed at 500 °C for 6 h. After the 36 h solvothermal process, the whole autoclave was kept inside the oven at room temperature for 6 or 24 h (referred to as aging, and hence the resultant product is named as aged LTO). Subsequently, the obtained sample was centrifuged, washed, and annealed at 500 °C for 6 h. The synthesis was repeated few times to evaluate the fabrication consistency of the aged LTO sample. For comparison, a sample with a ratio of 3.5:5 was fabricated through the aging process, denoted as aged LTO-3.5.

### Structural and Electrochemical Characterizations

The crystal structures and phases of the synthesized LTO-based nanostructures were identified by using X-ray diffraction (XRD; Rigaku Ultimate IV, Japan) and Raman spectroscopy (WITec Alpha 300R, Germany). Low-magnification and high-resolution imagings and selected-area electron diffraction (SAED) were carried out using transmission electron microscopy (TEM; TECHNAI, FEI, The Netherlands). X-ray photo-electron spectroscopy (XPS; Kratos, Axis Ultra, UK) measurements were taken to obtain surface information of the sample. The electrochemical performances of the synthesized nanostructures were evaluated by fabricating electrodes by slurry casting with an active material/CNT/polyvinylidene fluoride ratio of 75:15:10 (except for the rate test up to 1200*C* carried out with a ratio of 70:20:10 in comparison with the literature). The electrode loading was maintained in the range of 1 ± 0.2 mg cm^−2^. The cells were fabricated inside an Ar-filled glove box (O_2_ and H_2_O maintained below 1.0 part per million) either as coin cells (CR 2032) or Swagelok cells (diameter: 0.5 inch) with Li as the counter electrode in a half-cell assembly and electrolyte of 1 M of LiPF_6_ in ethylene carbonate/dimethyl carbonate. The half-cell electrochemical analysis was carried out in a voltage window of 0.8–3 V using an eight-channel battery cycler (BioLogic, USA). Li-ion battery full cells were fabricated by coupling the aged LTO sample with a LiMn_2_O_4_ (LMO) cathode in an anode-limited assembly cycled in a potential window of 1.5–3 V.

## Results and Discussion

**Fig. 1:**
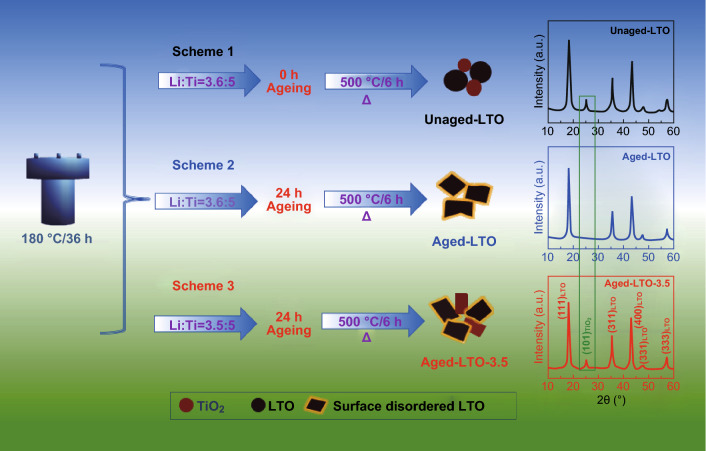
Solvothermal Schemes 1–3 with the physical and chemical characteristics of the unaged LTO, aged LTO, and aged LTO-3.5 samples and their XRD patterns

The synthesis of the surface-engineered spinel LTO by the solvothermal process is illustrated in Fig. 1. Scheme 1 describes the Li-deficient (ratio of Li/Ti of 3.6:5 against the nominal 4:5) process, which leads to LTO nanoparticles along with phase separation and crystallization of anatase TiO_2_. The XRD data are presented in the right panel of Fig. 1. The relative phase (weight) fractions of the components (LTO and TiO_2_) in the sample were quantitatively calculated by using the XRD pattern and Klug’s equation (Eq. 1):

1 $$W_{a} = {{I_{a} } \mathord{\left/ {\vphantom {{I_{a} } {\left( {I_{a} + \frac{{I_{b} }}{{K_{a}^{b} }}} \right)}}} \right. \kern-\nulldelimiterspace} {\left( {I_{a} + \frac{{I_{b} }}{{K_{a}^{b} }}} \right)}}$$

where *W*_*a*_ is the weight fraction of component “*a*” (LTO) in the mixed sample of LTO and TiO_2_, *I*_*a*_ and *I*_*b*_ are the integrated intensities of the highest peaks of components “*a*” and “*b,*” respectively, and $K_{a}^{b}$ (Eq. 2) is the ratio of the mass attenuation coefficients of components “*b*” and “*a*”:

2 $$K_{a}^{b} = R_{b} /R_{a} .$$

The mass attenuation coefficients of LTO (*R*_*a*_) and TiO_2_ (*R*_*b*_) are 103.94 and 118.65 cm^2^/g, respectively. *I*_*a*_ and *I*_*b*_ of the unaged LTO sample were 444.54 and 79.96, respectively. The weight fraction of component “*b*” (*W*_*b*_, Eq. 3) is

3 $$W_{b} = 1 - W_{a} .$$

Using these equations, the weight proportion of LTO/TiO_2_ in the unaged LTO sample was calculated to be 84:16.

Scheme 2 describes the aging at the end of the 36 h solvothermal process, which inhibited the TiO_2_ phase separation and crystallization, as confirmed by the XRD pattern, showing the absence of TiO_2_. Scheme 3 illustrates the synthesis with a decreased Li concentration (or increased Li deficiency) and aging leading to a phase-separated and crystalized TiO_2_ along with LTO. The XRD analysis confirmed that the TiO_2_ weight fraction was approximately 7 wt% (against the expected 20 wt%) [18]. This could be attributed to the aging enabling a certain weight fraction of TiO_2_ to self-assemble on the surface of LTO, while the remaining fraction was phase-separated and crystalized, which indicates that the surface layer thickness was possibly self-limited. The anatase TiO_2_ (101) peak at 24° was observed in the XRD pattern (not observed for Scheme 2).

A Raman analysis was carried out to investigate the phases in the samples. Figure S1a shows the Raman spectra of the aged and unaged LTO samples. The unaged LTO sample exhibited peaks matching with both LTO and TiO_2_. However, the *E*_g_ peak was shifted to a larger wave number, which can be associated with two factors, (1) phonon confinement and (2) surface strain induced by the oxygen deficiency in the lattice [19, 20]. The TiO_2_ signature was absent in the aged LTO, which confirms the absence of crystalline TiO_2_ particles in the sample. The 144 cm^−1^ (*E*_g_) vibration mode of TiO_2_ is very sensitive and is typically observed even for a small quantity of TiO_2_. The Raman analysis is consistent with the above XRD results, which confirms the presence and absence of TiO_2_ in the unaged and aged LTO samples, respectively. High-resolution Ti 2*p* XP spectra of both samples are presented in Fig. S1c. Both samples exhibited similar profiles with a binding energy of 458.4 eV, which suggests the presence of octahedrally coordinated Ti species. The spin–orbit coupling of 5.7 eV confirms the existence of Ti in the 4+ oxidation state. As both LTO and TiO_2_ have Ti in the 4+ oxidation state, it is challenging to independently identify their presence (as the XPS peaks overlay at the same binding energies). However, a small increase in peak width (~ 0.2 eV) was observed for the aged LTO sample, which could be originated from the formation of a disordered surface layer. Figure S1c shows a survey XP spectrum of the aged LTO sample, which confirms the absence of other surface impurities in the sample.

**Fig. 2:**
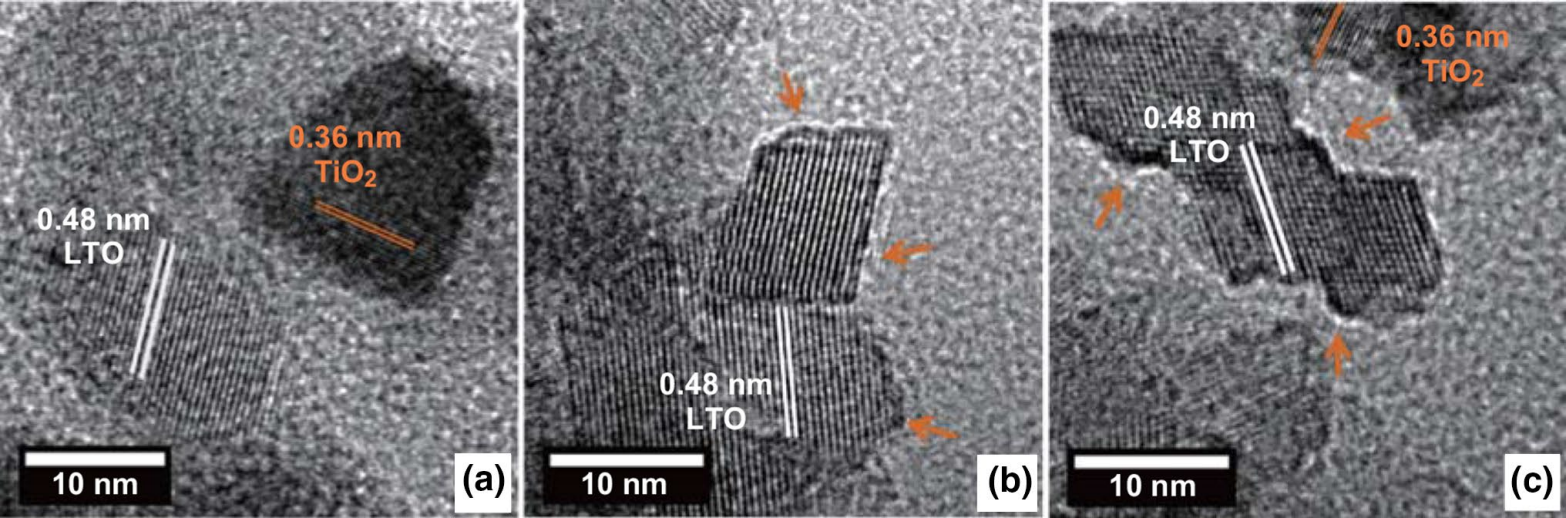
High-resolution TEM analysis of the fabricated materials. **a** Unaged LTO with both LTO and TiO_2_ phases, **b** aged LTO with a disordered surface layer (indicated by arrows), and **c** aged LTO-3.5 with both disordered surface layer (indicated by arrows) and TiO_2_ phase

The TEM results confirm the crystalline structures of all three samples, with average particle sizes of approximately 20 nm (Fig. 2a–c). This confirms that the aging did not lead to Ostwald ripening, but the particle size was preserved even after the aging. In addition, three striking features were observed in the TEM images. (1) The unaged sample contained both LTO and TiO_2_, (2) the aged LTO sample exhibited only an LTO plate-like morphology with a disordered surface layer, and (3) the aged LTO-3.5 contained a plate-like LTO with a disordered surface layer as well as TiO_2_. These observations are consistent with the XRD results in Fig. 1. Notably, the unaged and aged samples exhibited similar morphologies before the annealing (Fig. S2a, b). However, after the annealing, the unaged sample exhibited a particle-like morphology, while the aged sample retained the plate-like morphology, as shown in the TEM images (Fig. 2a, b). Additional TEM images showing the surface disordered layer and plate-like morphology of the aged LTO sample are presented in Fig. S2c, d for reference. Figure S3 shows SAED patterns, which confirms the coexistence of crystalline LTO–TiO_2_ phases in the unaged sample and only spinel LTO phase in the aged LTO sample, consistent with the XRD results. To correlate the morphology changes of the unaged and aged samples with the phase changes, XRD patterns were recorded before the annealing in comparison with those after the annealing. Before the annealing, all samples exhibited identical patterns (Fig. S4) with broad peaks, which could be indexed to the orthorhombic lithium titanate hydrate phase (JCPDS No. 00-047-0123). Upon the annealing, the unaged sample crystallized to LTO–TiO_2_ (dual phase), while the aged sample crystallized to LTO (single phase). The formation of such a disordered surface layer could be explained as follows. Upon the completion of the solvothermal reaction, the lithium titanate hydrate phase formed in the Li-deficient precursor ratio. Annealing the solveothermal product immediately led to phase-separated crystalline TiO_2_ particles (dual phase) in the unaged LTO sample. However, before annealing, if these nanostructures are allowed to age, self-assembling could occur on the surface of LTO, which forms a disordered thin layer without crystallization of TiO_2_. For the aged LTO sample, this is confirmed by the absence of crystalline TiO_2_ according to both XRD and TEM analyses. The aging requires approximately 24 h. In this regard, another sample subjected to 6 h of aging was analyzed. It still exhibited the LTO–TiO_2_ dual phase (weight proportion of LTO/TiO_2_ = 88/12) with a very small decrease in TiO_2_ fraction compared to that of the unaged sample (Fig. S5). This indicates that a larger aging time is required to inhibit the TiO_2_ phase separation and crystallization from the hydrated phase.

**Fig. 3:**
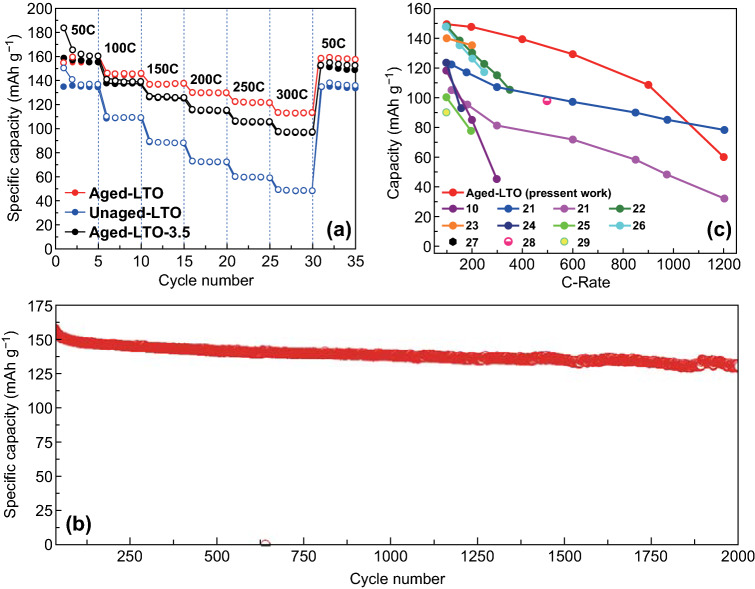
Electrochemical performances of the half cells. **a** Ultrahigh-rate performances of the aged and unaged LTO electrodes at rates shown in the legend. **b** Long-cycling performance of the aged LTO sample at 50*C*. **c** Rate performance of the aged LTO sample up to 1200*C* and high-rate LTO literature values

To evaluate the electrochemical performances of the surface-engineered nanostructures, we carried out a rate test (Fig. 3a) in the range of 50*C* to 300*C* (charge and discharge for five cycles at each rate). The aged LTO sample exhibited an exceptionally high performance with a value of 155.9 mAh g^−1^ at 50*C* and retained 72% of the initial capacity even at 300*C* with a Coulombic efficiency of almost 100%. According to the charge–discharge profiles in Fig. S6, the voltage plateau even at such ultrahigh rates indicates that the material underwent bulk lithiation/delithiation rather than mere surface/interface storage. The charge–discharge voltage polarization of the aged LTO sample was smaller than that of the unaged LTO. The lower polarization indicates that the CNT additive formed a percolated conductive pathway with the smallest resistance for the electron transport across the thickness of the electrode. Even upon reversion from 300*C* to 50*C*, the aged LTO electrode retained 100% of the value at the initial 50*C* cycles, which demonstrates the excellent reversibility and stability of the sample. The aged LTO-3.5 sample exhibited an inferior performance, though at 50*C* the performance was similar to that of the aged LTO; at 300*C*, the specific capacity was 156 mAh g^−1^. On the other hand, the unaged LTO sample exhibited a considerably lower performance, only 136 mAh g^−1^ at 50*C* and only 49 mAh g^−1^ at 300*C*. The specific capacity of the aged LTO at 300*C* was 134% higher than that of the unaged LTO sample. This difference is also reflected in the electrochemical impedance spectra (EIS) of cycled cells consisting of both samples, presented in Fig. S7. The lower charge-transfer resistance of the aged LTO can be related to the better transport of ions across the surface disordered layer. The diffusion coefficient of the aged LTO, calculated by using the EIS, was 0.75 × 10^−9^ cm^2^ s^−1^, comparable or higher than those in the literature, mostly in the range of 10^−9^ to 10^−13^ cm^2^ s^−1^ [1, 14]. However, diffusion coefficients higher than those in this study have been reported [6]. The better diffusion coefficient could be attributed to the plate-like morphology promoting a better diffusion of Li ions. The electrochemical results demonstrate that ultrahigh-rate (300*C*) capabilities (both charge and discharge within 12 s) can be achieved for a lithium-ion battery electrode while maintaining the high specific capacity.

Figure 3b shows the long-cycling performances of the aged LTO samples cycled at a charge/discharge rate of 50*C*. The electrode delivered a first-cycle specific capacity of 156 mAh g^−1^ and retained 82.6% of the initial capacity at the 2000th cycle. In addition, the electrode provided a charge capacity of 156 mAh g^−1^ and retention of approximately 70% at 100*C* (discharge limited to 50*C*) for 2000 cycles (Fig. S8). Further, we investigated the ultrahigh-rate capability of the aged LTO through a rate test beyond 300*C*. Figure 3c shows the rate performance of the aged LTO electrode (100*C*–200*C*–400*C*–600*C*–900*C*–1200*C*; all discharge rates were limited to 100*C*). The charge–discharge profiles are presented in Fig. S9. The electrode delivered specific capacities of 149 mAh g^−1^ at 100*C* and 129 mAh g^−1^ at 600*C*. The obtained results are compared to ultrahigh-rate (100*C* or above) data reported in the literature in Fig. 3c [10, 21–29]. At the maximum charge rate of 1200*C* (theoretically, equivalent to 100% delithiation in 3 s), the electrode delivered almost 60 mAh g^−1^. The ultrahigh-rate supercapacitor-like battery performance of the aged LTO electrode confirms the potential of the surface engineering strategy for the fabrication of lithium-ion battery electrodes for high-power battery applications. We also compared the electrochemical performances of the aged LTO to those of other high-rate lithium-ion battery electrode materials (Table S1) [17, 30–38].

The formation of the defective surface layer in the aged LTO enabled a faster charge transfer than those of the unaged LTO and even aged LTO 3.5. Moreover, in combination with the surface layer enabling the better charge transfer, the smaller particle size and nanoplate morphology led to a high diffusion coefficient. Additionally, the blending with the CNT additive provided a better electrode conductivity. Thus, by combining the principles of nanoengineering (synthesis of LTO nanoplates), surface engineering (formation of a defective surface layer), and electrode engineering (aged LTO composited with the high-aspect-ratio CNT additive), a battery with an ultrahigh-rate capacity, high cycling stability, and wide temperature range of operation was fabricated. In other words, the aged LTO sample addresses the above three aspects, which limit the rate performance of LTO, owing to the (1) synthesis of the nanoplate-like structure and improved diffusion kinetics, (2) electrode engineering by compositing with the CNT additive, and (3) surface engineering by creating a disordered surface layer, which facilitated the charge transfer at the electrode–electrolyte interface.

Considering the superior performance of the electrode, we also investigated its practical use by evaluating the effect of the active material loading through the delivered areal capacity. Figure S10 shows a high areal capacity of 0.6 mAh cm^−2^ at 10*C* (loading: 3.56 mg cm^−2^) for 100 cycles. A detailed analysis is presented in Supplementary Material. Moreover, we investigated the structural stability of the aged LTO sample by ex situ TEM imaging on an electrode cycled at a rate of 10*C* for 250 cycles. As shown in Fig. S11, the particles retained the surface-coated plate-like structure even after the long cycling, which demonstrates the structural stability.

**Fig. 4:**
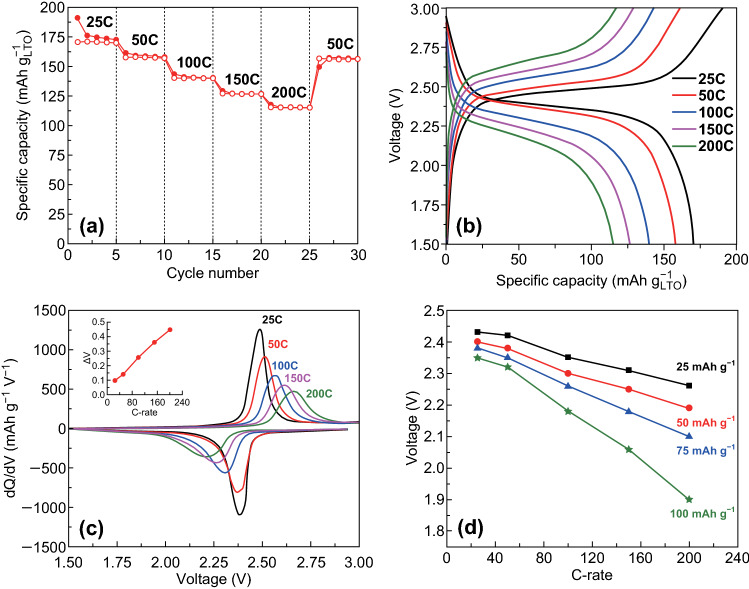
Electrochemical performances of the full cells. **a** Rate performance of the aged LTO/LMO full cell at high rates of 25*C* to 200*C*. **b** First-cycle charge–discharge profile of the fabricated aged LTO/LMO full cell. **c** d*Q* d*V*^−1^ plots of the full cell at different rates (the inset shows the voltage polarization as a function of the *C*-rate). **d** Measured discharge voltages at different rates and different states of discharge corresponding to 25, 50, 75, and 100 mAh g^−1^

To demonstrate the potential of the aged LTO for use in an ultrafast-charging battery, a full cell was fabricated by coupling the aged LTO with a spinel LMO cathode [39]. An anode-limited full cell was fabricated, and the cycling rates, specific capacity, and energy/power densities were calculated with respect to the active weight of the aged LTO [40]. Figure 4a shows the rate performance of the full cell in the voltage window of 1.5 to 3.0 V. The full cell exhibited discharge capacities of 170, 157, 140, 127, and 115 mAh g^−1^ at ultrahigh rates of 25*C*, 50*C*, 100*C*, 150*C*, and 200*C*, respectively, with respect to the LTO electrode. The ultrahigh-rate lithium-ion battery is beneficial for practical applications such as electric vehicles for fast charging and even for on-route charging. The first-cycle charge–discharge profiles of the full cell at different rates are shown in Fig. 4b. At 200*C*, the power and energy densities of the full cell were 76 kW kg^−1^ and 249 Wh kg^−1^, respectively (Table S2), which demonstrates the potential of the aged LTO-based full cell for high-power applications. Considering the theoretical specific capacity of the aged LTO, 65% of the theoretical capacity was delivered in 12 s (at the rate of 200*C*). Figure 4c shows d*Q* d*V*^−1^ of the full cell at different rates, as indicated in the legend. Sharp peaks of the full cell were observed even at rates as high as 200*C.* The inset shows the voltage polarization as a function of the *C*-rate, which shows a linear increase indicating a good rate capability. The voltage polarization was symmetric (at all rates) in the lithiation/delithiation, which indicates a fast intercalation/deintercalation of lithium. Figure 4d shows the measured discharge voltages at different states of discharge corresponding to specific capacities of 25, 50, 75, and 100 mAh g^−1^. The smooth and linear voltage drop of the full cell confirms that the overvoltage was smaller than 0.5 V for the increase in *C*-rate from 25*C* up to 200*C*. This was achieved by both electrode engineering at large scales and surface engineering at the nanoscale. These results show the excellent performances of the full cell at high charge–discharge rates reported in the literature so far.

**Fig. 5:**
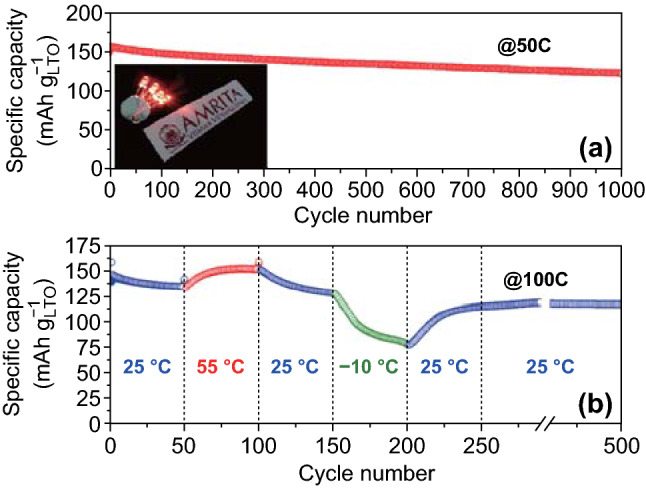
Electrochemical cycling stability and operation temperature of the full cell. **a** Long-cycling performance at 50*C* (both charge and discharge). **b** Performances at different temperatures (shown in the legend) for cycling at 100*C*. The inset in (**a**) shows that the four light-emitting diodes could be lit by the coin cell, which demonstrates the high-power capability

In addition to the ultrahigh-rate capability, the full cell also exhibited a high cycling stability for 1000 cycles with a retention of approximately 82% at 50*C* (after the rate test), as presented in Fig. 5a. As shown in the inset, four high-power light-emitting diodes could be lit with a single coin cell, which shows its power-delivering capability. Figure S12 presents results for the full cell consisting of the aged LTO-3.5 and LMO cathode, which exhibited a reasonably high-rate performance, though inferior to that of the aged LTO full cell. To evaluate the full-cell high-rate performances in a wide temperature range, the full cell (aged LTO and LMO) was tested at room temperature (25 °C), high temperature (55 °C), and low temperature (− 10 °C). Figure 5b shows the specific capacity as a function of the cycle number for the full cell operated at various temperatures for 50 cycles (at each temperature, as indicated in the legend) at 100*C*. The data show that the full cell retained almost 150 mAh g^−1^ at the high temperature and above 75 mAh g^−1^ at the low temperature. After the thermal cycles and reversal to room temperature, the full cell was stable over 500 cycles and retained a capacity of 116 mAh g^−1^. Although LTO/LMO full cells have been investigated by different groups, we report improved results in terms of rate capability and corresponding specific capacities [41–44]. The proposed structure is one of the safest electrode combinations with potentials for use in high-end electric vehicle applications [45, 46].

## Conclusions

Surface-engineered LTO nanostructures fabricated through the off-stoichiometric (Li-deficient) solvothermal process and following aging exhibited an ultrahigh-rate capability. The aging had significant effects; it not only inhibited the TiO_2_ phase separation and crystallization, but also provided a self-assembled disordered surface layer on LTO. The further increase in Li deficiency led to the phase separation of TiO_2_ apart from the formation of the surface disordered layer, which indicates that its thickness was self-limited. The aged LTO plates as an anode for the Li-ion battery provided excellent electrochemical performances in terms of specific capacity, cycling life, and ultrahigh-rate capability. The electrode exhibited high specific capacities of 129 mAh g^−1^ at the high rate of 600*C* and 60 mAh g^−1^ at 1200*C*. Further, the aged LTO/LMO full cell exhibited a fast charging–discharging (up to 200*C* with a time equivalent of 12 s) and long-cycling capability without affected capacity and nominal voltage. The high diffusion coefficient enabled the improved kinetics, the CNTs enabled the good electrode conductivity, and the surface disorder layer enabled the better charge transfer, which led to the ultrahigh-rate performance and cycling stability in the wide temperature range. This demonstrates that the control of the surfaces of the nanomaterials could enable high-power Li-ion batteries. This is a commercially viable strategy and can even be extended to other electrode materials to materialize the ultrahigh-power lithium-ion battery chemistries.

## Electronic supplementary material

Below is the link to the electronic supplementary material.

Supplementary material 1 (PDF 1291 kb)
