# Supplementary material for: Surface-Engineered Li4Ti5O12 Nanostructures for High-Power Li-Ion Batteries
Source: Nanomicro Lett. 2020 Jan 21;12:30. doi: 10.1007/s40820-020-0366-x (PMC7770703; doi:10.1007/s40820-020-0366-x)
Supplement: Supplementary file 1 — Supplementary material 1 (PDF 1291 kb) [file 40820_2020_366_MOESM1_ESM.pdf]

Supporting Information for

Surface-Engineered $Li_4Ti_5O_{12}$ Nanoparticles for High-Power Li-Ion Batteries

Binitha Gangaja[1], Shantikumar Nair[1], Dhamodaran Santhanagopalan[1,] *

[1]Centre for Nanosciences and Molecular Medicine, Amrita Vishwa Vidyapeetham, AIMS (P.O), Kochi – 682 041, India

*Corresponding author. E-mail: dsgopalan20710@aims.amrita.edu (Dhamodaran Santhanagopalan)

Supplementary Figures and Tables

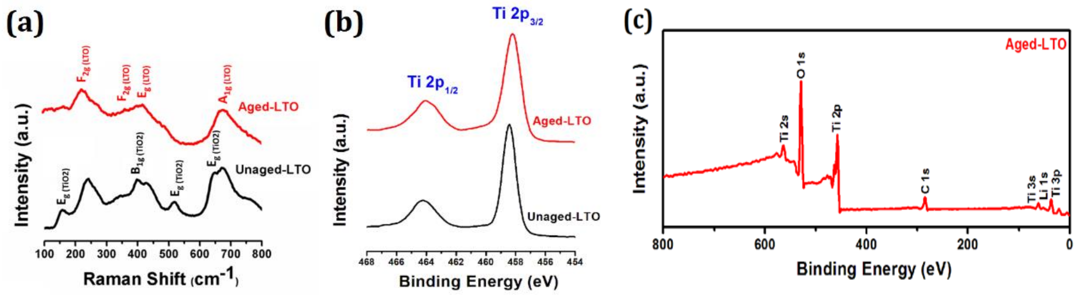

Fig. S1 (**a**) Raman spectra of unaged and aged-LTO sample (**b**) XPS high resolution Ti 2p spectra of aged and unaged-LTO samples and (**c**) XPS survey spectra of Aged-LTO showing absence of other impurities

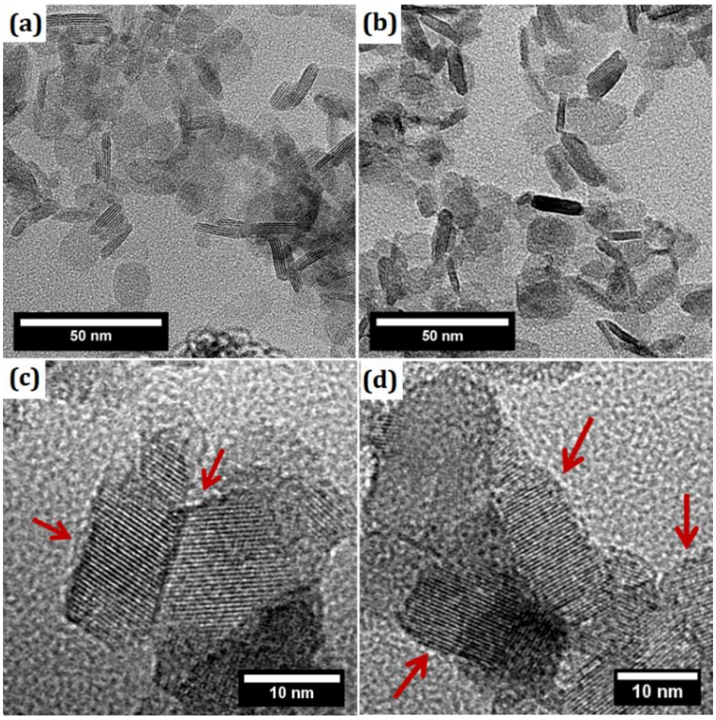

Fig. S2 TEM images at before annealing condition of **(a)** Unaged-LTO (**b**) Aged-LTO. (**c**, **d**) High magnification TEM image of the aged-LTO sample with surface disordered layer indicated in red arrows

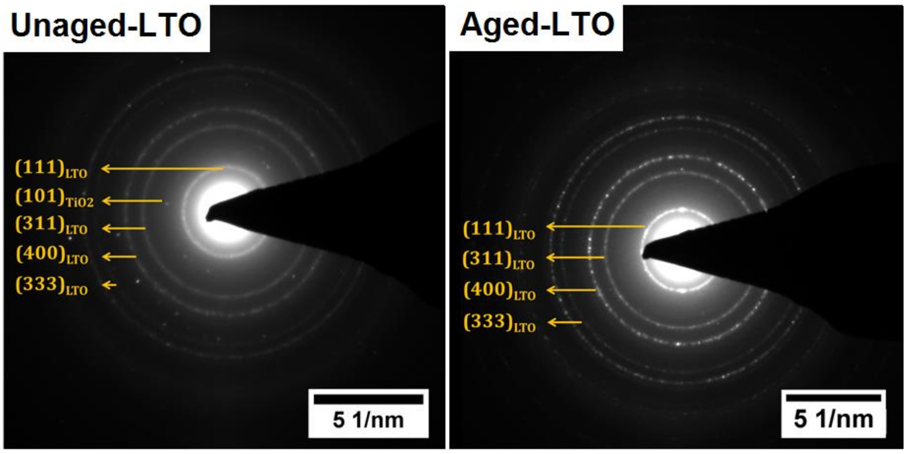

Fig. S3 Selected area diffraction pattern of both the samples

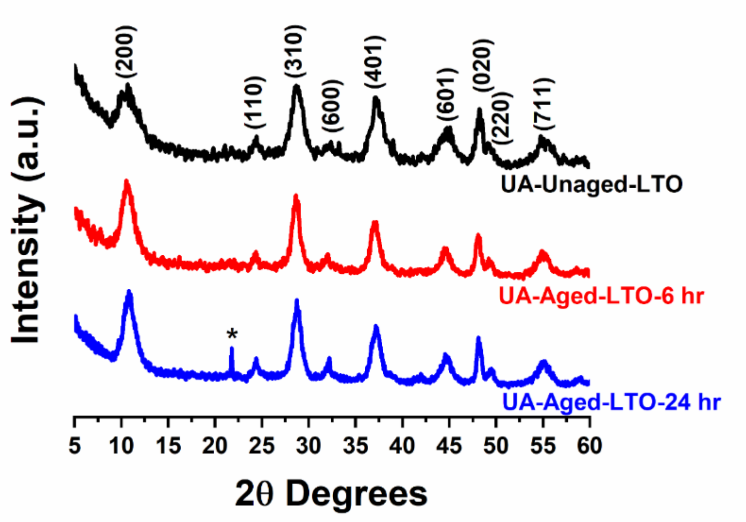

Fig. S4 X-ray diffraction pattern of unannealed samples

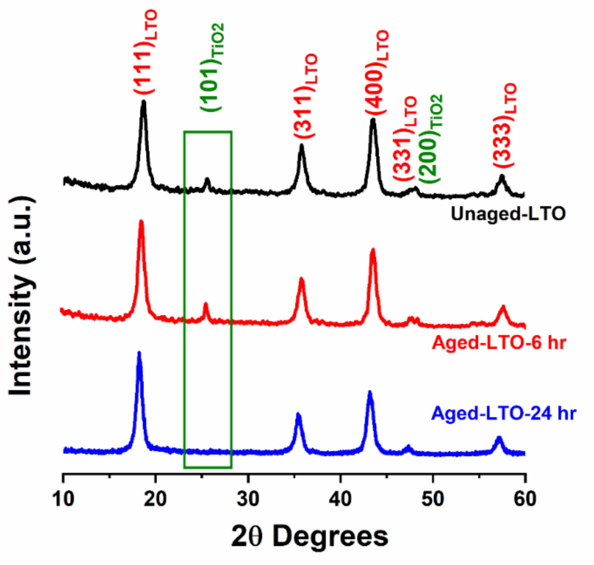

Fig. S5 X-ray diffraction for annealed samples of unaged-LTO, aged-LTO-6 h and aged-LTO-24 h samples

Based on the previous reports [S1, S2], and XRD datas of the present work, unannealed samples have been identified to be orthorhombic lithium titanate hydrate phase (JCPDS# 00-047-0123) irrespective of ageing time (0, 6, 24 h). Due to lithium deficiency layered hydrated titanium oxide with a general formula of $H_2Ti_nO_{2n+1}$ may also form in unannealed samples (as prepared) [S1]. This hydrate phase depending on the annealing temperature, converts into different hydrated phases but for temperatures as high as 500 °C upon dehydration, it crystalizes into LTO and TiO_2 [S1]. However, in the present work we have shown that providing a critical aging time (24 h) would inhibit the phase separation and crystallization of TiO_2 (due to lithium deficienct precursor) and forms as a disordered layer on the surface of LTO. Amorphous phase of TiO_2 have been reported to be stable even up to 700 °C without any structural evolution [S3].

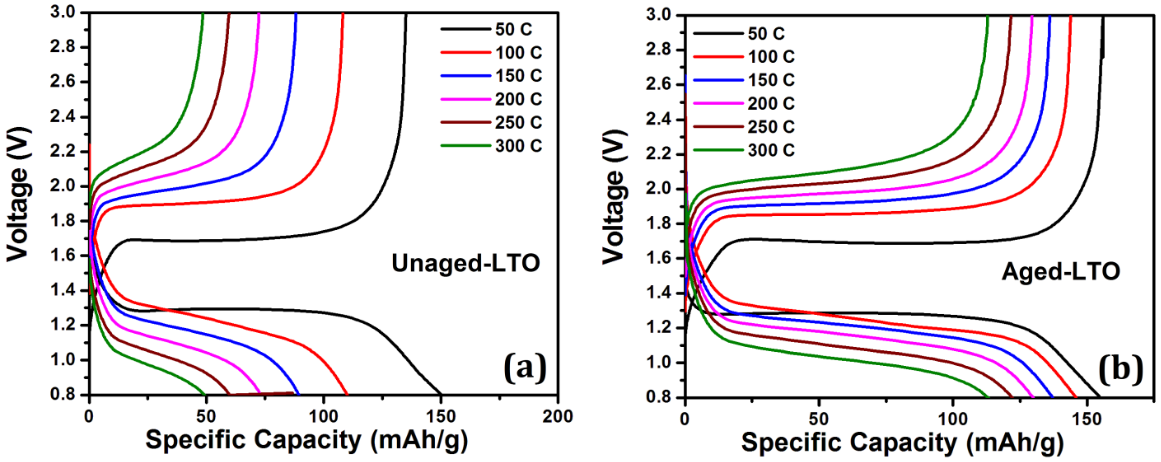

Fig. S6 Charge-discharge profiles at rates ranging from 50C to 300C of (**a**) unaged-LTO and (**b**) aged-LTO

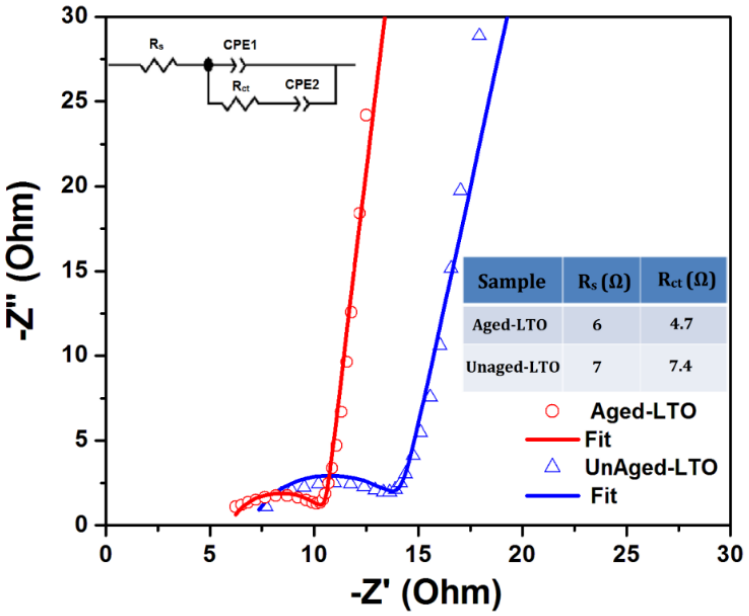

Fig. S7 Electrochemical impedance spectra of both samples after cycling at 150C for 100 cycles. Inset shows the equivalent circuit and fitted resistance values

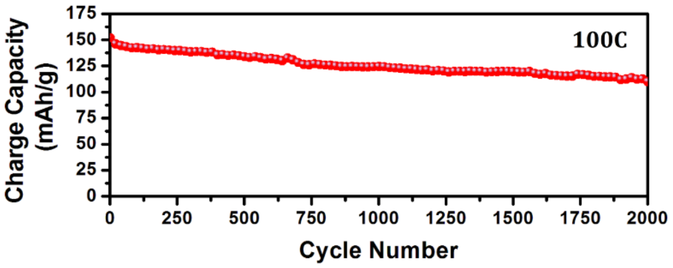

Fig. S8 Long cycling performance of aged-LTO sample at a rate of 100C

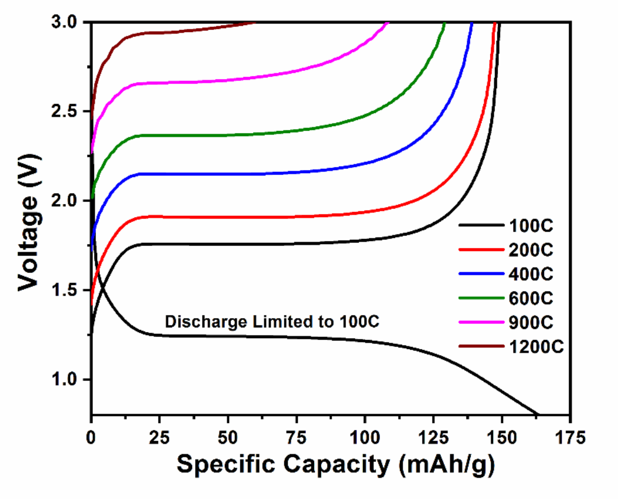

Fig. S9 Charge profiles of the Aged-LTO sample at different rates wherein the discharge was limited to 100C

Table S1 Electrochemical performance comparison of aged-LTO with the high rate performing lithium ion battery electrodes

Sl. No.	Active Material	Theoretical capacity (1C equivalent) (mAh g^{-1})	Maximum Reported Rate (C or mA g^{-1})	Capacity delivered (mAh g^{-1})	Refs.
1	$LiFePO_4$	166	397C	60	[17]
2	Black TiO_2	200	100C	152	[30]
3	Nb_2O_5 Holey graphene oxide	404	100C	75	[31]
4	C-coated $LiMn_2O_4$	120	300C	~57	[32]
5	Sb doped SnO_2/graphene	1494	60C	88	[33]
6	Lithiated MnO_2	-	1114C	75	[34]
7	3D printed $LiMn_{1-x}Fe_xPO_4$	-	100C	108	[35]
8	$FePO_4$	170	600C	72	[36]
9	PTMA-Graphene	222	200C	80	[37]
10	N-doped graphene<br>B- doped graphene	200	25 A/g	199<br>235	[38]
11	Aged-LTO	175	50C<br>300C<br>1200C	156<br>112<br>60	This work

Beyond the superior performance of electrodes, we have also investigated the practical use the electrode by tested the effect of active material loading through delivered areal capacities. Figure S10a shows the high areal capacity of about 0.6 mAh cm^{-2} delivered by aged-LTO at 10C rate for 100 cycles. This compares well with commercial electrodes that deliver about 1.0 mAh cm^{-2} at slow rates while active material coated on both sides of the current collector. Figure S10b shows the areal capacity as a function of cycling number for aged-LTO electrode with varying active materials loading at 50C rate. It is witnessed that the electrodes active weight ranging from 0.964 to 2.785 mg cm^{-2} delivers high areal capacity of about 0.15 to 0.35 mAh cm^{-2} respectively at 50C for 100 cycles with high stability. The inset in Fig. S10a shows almost linear variation of areal capacity with active loading indicating excellent electro-chemical performance with loading. Thus the high areal capacity retained at 50C by aged-LTO establishes its use as anode in ultra-fast applications.

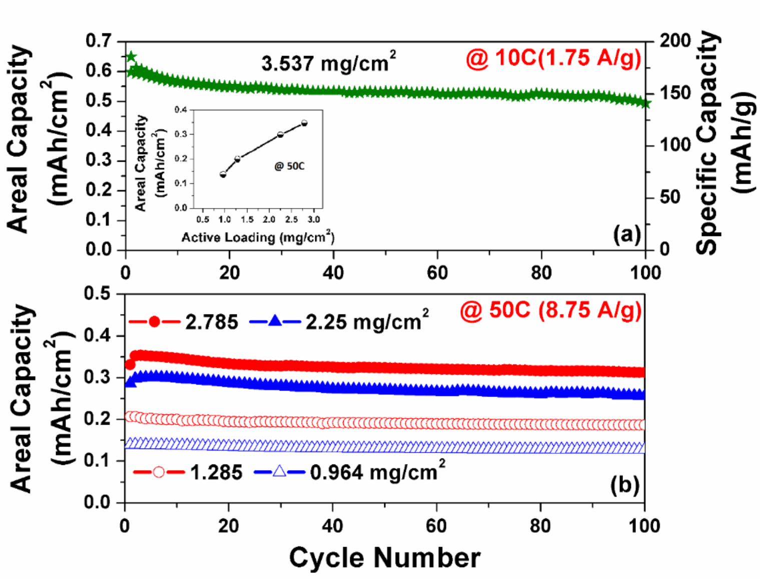

Fig. S10 (**a**) Areal capacity of Aged-LTO half-cell at 10C rate for 100 cycles and (**b**) areal capacity of the aged-LTO electrode at 50C rate (with different active material loading). Inset in (**a**) shows the areal capacity increases almost linearly with loading

Table S2 Electrochemical performance details of aged-LTO/LMO full-cell with the energy density and power density values

Rate	Voltage at 50% Capacity	Discharge Capacity (mAh/g_{LTO})	Discharge Time (s)	Energy Density (Wh/kg)	Power Density (kW/kg)
25	2.36	170.28	139.9	401.9	10.34
50	2.34	157.36	64.75	368.2	20.47
100	2.27	140	28.8	317.8	39.73
150	2.2	126.55	17.34	278.4	57.78
200	2.16	115.18	11.85	248.8	75.58

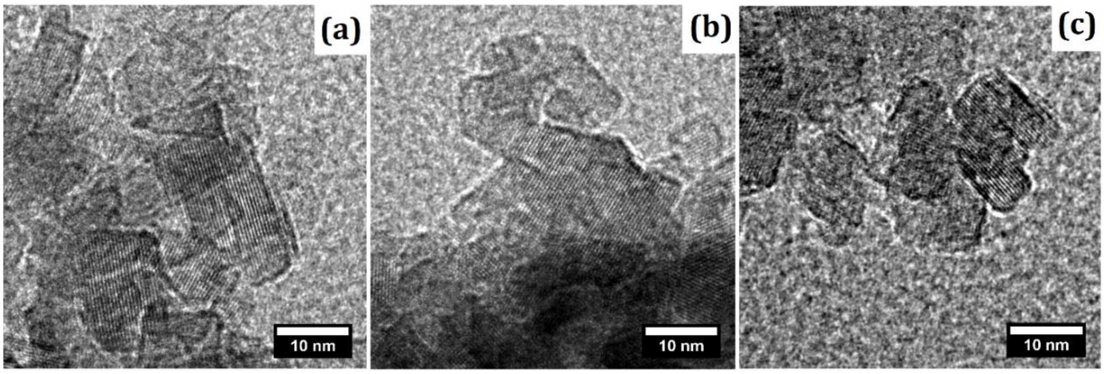

Fig. S11 a-c *ex situ* TEM images of cycled Aged-LTO electrode at 10C for 250 cycles

We have also tested the full-cell performance of aged-3.5 sample by coupling with the LMO cathode. Figure S12 a, b shows the rate performance and 1st cycle charge-discharge profile of the full-cell at different rates ranging from 50C-200C. At 50C the full-cell delivered 112 mAh g^{-1} while around 70 mAh g^{-1} discharge capacity was exhibited at 200C. Further the aged-3.5/LMO full-cell demonstrated good cycling stability (Fig. S12c) for 1000 cycles with discharge capacity slightly less as compare to the aged-LTO/LMO full-cell. From this full-cell data it can be confirmed that surface engineered aged-LTO sample has better deliverable capacity at high rates as compared to the TiO_2 phase separated aged-3.5/LMO full-cell.

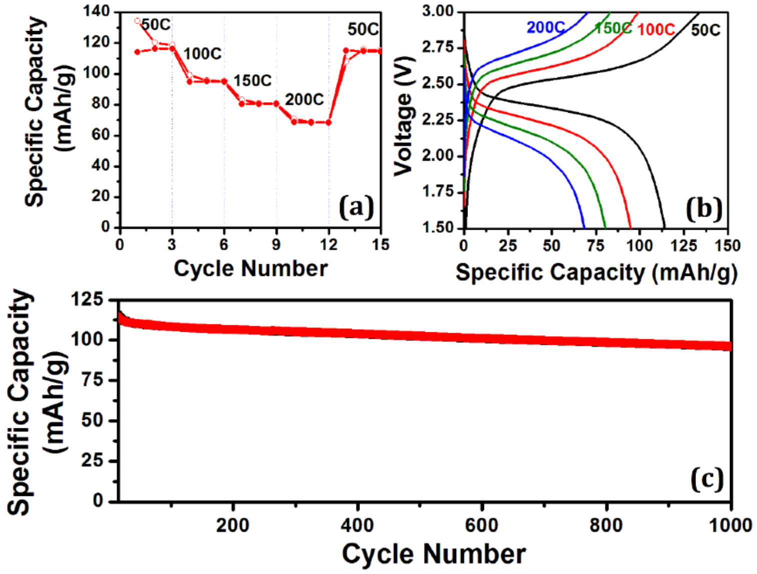

Fig. S12 (**a**) Rate performance of aged-3.5/LMO full-cell at high rates from 50C to 200C, 3 cycles each (**b**) 1st cycle charge-discharge profile fabricated aged-3.5/LMO full-cell and (**c**) long cycling performance of the lithium ion battery full-cell at 50C rate (both charge and discharge) for 1000 cycles

Supplementary References

[S1] S. Wang, W. Quan, Z. Zhu, Y. Yang, Q. Liu et al., Lithium titanate hydrates with superfast and stable cycling in lithium ion batteries. Nat. Commun. **8**, 627 (2017). https://doi.org/10.1038/s41467-017-00574-9

[S2] Z.S. Hong, M.D Wei, Layered titanate nanostructures and their derivatives as negative electrode materials for lithium-ion batteries. J. Mater. Chem. A **1**, 4403 (2013). https://doi.org/10.1039/c2ta01312f

[S3] M. Hannula, H.A. Loytty, K. Lahtonen, E. Sarlin, J. Saari, M. Valden, Improved stability of atomic layer deposited amorphous TiO2 photoelectrode coatings by thermally induced oxygen defects. Chem. Mater. **30**, 1199 (2018). https://doi.org/10.1021/acs.chemmater.7b02938
